# Supplementary material for: Formaldehyde Emissions from Wooden Toys: Comparison of Different Measurement Methods and Assessment of Exposure
Source: Materials (Basel). 2021 Jan 7;14(2):262. doi: 10.3390/ma14020262 (PMC7825799; doi:10.3390/ma14020262)
Supplement: Supplementary file 1 [file materials-14-00262-s001.pdf]

materials

MDPI

Supplementary Materials

Formaldehyde Emissions from Wooden Toys: Comparison of Different Measurement Methods and Assessment of Exposure

Morgane Even [1,2,3,*], Olaf Wilke [2], Sabine Kalus [2], Petra Schultes [4], Christoph Hutzler [1] and Andreas Luch [1,3]

[1] German Federal Institute for Risk Assessment (BfR), Department of Chemical and Product Safety, Max-Dohrn-Strasse 8-10, 10589 Berlin, Germany; christoph.hutzler@bfr.bund.de (C.H.); andreas.luch@bfr.bund.de (A.L.)
[2] Bundesanstalt für Materialforschung und -prüfung (BAM), Division 4.2–Materials and Air Pollutants, Unter den Eichen 44-46, 12203 Berlin, Germany; olaf.wilke@bam.de (O.W.); sabine.kalus@bam.de (S.K.)
[3] Freie Universität Berlin, Department of Biology, Chemistry, Pharmacy, Institute of Pharmacy, Königin-Luise-Strasse 2-4, 14195 Berlin, Germany
[4] Chemical and Veterinary Analytical Institute Münsterland-Emscher-Lippe (CVUA-MEL), Joseph-König-Str. 40, 48147 Münster, Germany; petra.schultes@cvua-mel.de
* Correspondence: morgane.even@bam.de

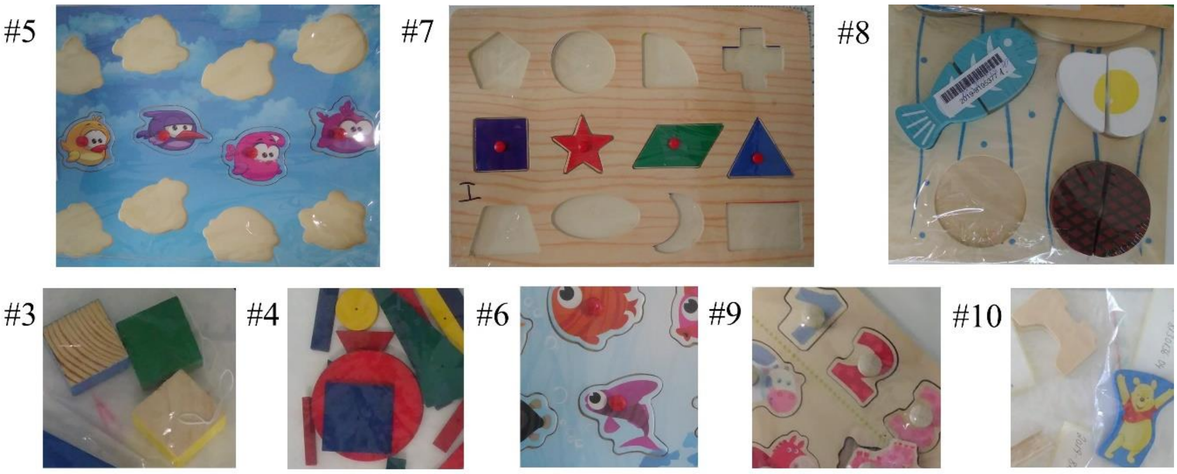

Figure S1. Representative pictures of the investigated toy samples.

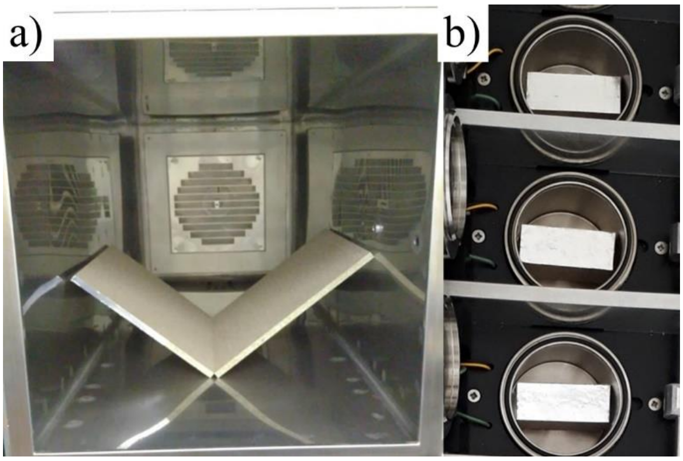

Figure S2. Example image of chamber loading for sample #1 in the 1 m³ chamber (**a**) and the microchamber (**b**).

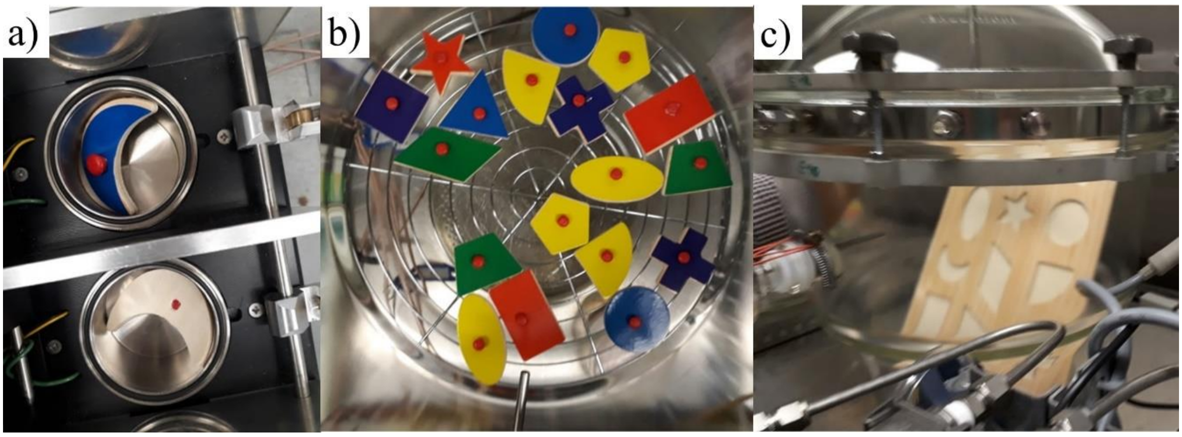

Figure S3. Example image of chamber loading for sample #7: Pieces in the microchamber (**a**), pieces in the desiccator (**b**) and plate in the desiccator (**c**).

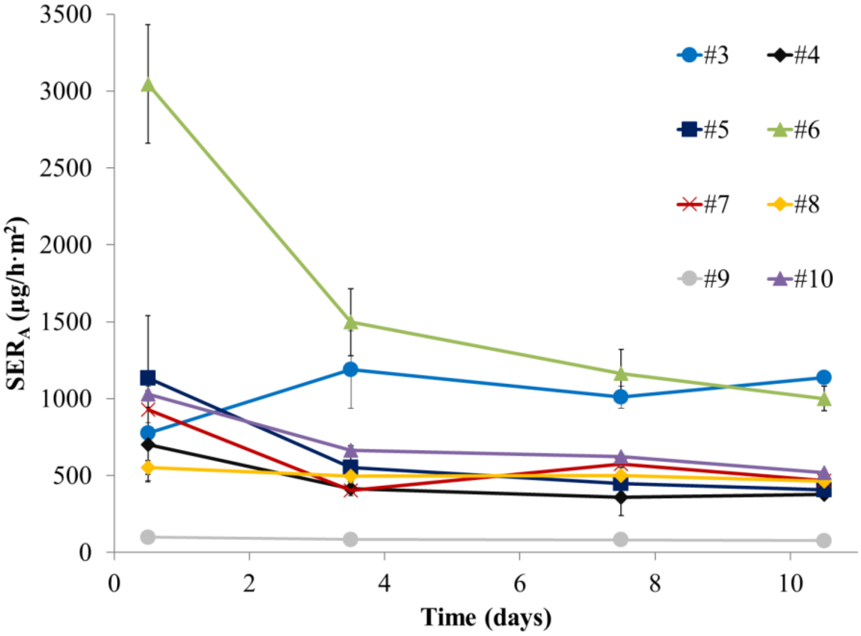

Figure S4. Emission profiles of formaldehyde from wooden toy pieces in the microchamber over 10 days (n = 2; SER_A, area-specific emission rate). Error bars represent standard deviation (SD).

Table S1. Dimensions of the samples for chamber studies; [¥]: sample edges were partly covered; *: approximated shape; ellipses, circles: 1st dimension are diameters.

Microchamber Samples

No.	Geometric Shape	1st Dimension (cm)	2nd Dimension (cm)	Face Surface Area (cm^2)	Perimeter (cm)	Thickness (cm)	Total Surface Sample Surface Area (cm^2)	Weight (g)
#1[¥]	parallelepiped	4.0	2.0	8.0		0.16	16.0	3.2/3.3/3.4
#2[¥]								3.3/3.4/3.6
#3	parallelepiped	2.97	2.99	8.9		1.40	34.4	5.5/6.2
#4	cylinder	4.03		12.8		0.27	28.9	1.8/2.3
#5[¥]	half ellipse*	4.40	2.30	8.3	8.0	0.37	19.7	1.3/1.4
#6[¥]	triangle*	4.55	2.20	5.0	8.0	0.39	12.6	1.6
#7	2 half ellipses*	5.00 and 4.20	5.00 and 1.80	9.0	14.3	0.41	24.0	2.1
#8[¥]	quarter circle*	5.54	86°	5.8	6.7	1.84	23.9	8.5
#9	ellipse*	4.07	3.50	11.2	13.8	0.48	29.0	8.3
#10	parallelepiped*	2.98	2.04	6.1	11.4	0.48	16.9	1.9

Dessicator Samples

No.	Number of Pieces	Total Pieces Surface Area (cm^2)	Number of Plates	Total Plates Surface Area (cm^2)
#5	16	651	1	1456
#7	18	711	1	1426
#8	8	828	2	1475

1 m^3 Chamber Samples

No.	Number of Plates	Total Plates Surface Area (cm^2)
#1[¥]	2	0.93
#2[¥]		

Table S2. Flask method values of the samples similar to those studied in the microchamber and of samples after microchamber testing (mg/kg$_{toy}$); usually, different pieces of the same toy sample were put together in the flask: the values reported here are the mean and standard deviation of the measurement with samples having the same characteristics (in particular color) than the samples studied in the microchambers; *: ±30% SD were estimated from experience of CVUA-MEL because there were no replicates.

No.	Similar Samples	After Microchamber (n = 1)
#1	116 ± 35* (n = 1)	not measured
#2	117 ± 35* (n = 1)	not measured
#3	310 ± 80 (n = 2)	346 ± 104*
#4	865 ± 21 (n = 2)	365 ± 110*
#5	250 ± 39 (n = 4)	309 ± 75*
#6	530 ± 27 (n = 3)	561 ± 158*
#7	345 ± 71 (n = 2)	286 ± 86*
#8	101 ± 13 (n = 3)	not measured
#9	47 ± 14* (n = 1)	not measured
#10	344 ± 103* (n = 1)	not measured
